# Adjustments of balance control during cognitive dual tasking: Evidence from event-related force-plate analysis

**DOI:** 10.1007/s00426-025-02215-z

**Published:** 2025-12-06

**Authors:** Anton Koger, Leif Johannsen, Andrea Kiesel, Hermann Müller, Denise Nadine Stephan, Elisa Ruth Straub, Iring Koch

**Affiliations:** 1 Institute of Psychology, RWTH Aachen University, Aachen, Germany; 2 Department of Psychology, University of Freiburg, Freiburg, Germany; 3 Department of Sports Science, University of Giessen, Giessen, Germany

## Abstract

**Abstract:**

Cognitive-motor interference refers to the interaction between cognitive and motor processes occurring at the same time. Recently, balance control parameters while standing on a force plate were analysed using an event-related approach while participants performed a Simon task. Resolving response conflict in incongruent trials reduced balance adjustments prior to manual response execution, suggesting a bottleneck for concurrent cognitive and balance control. In the present study, we combined this approach with a cognitive dual task which comprised a visual-vocal short-term memory task with a delayed vocal response and an auditory-manual reaction time (RT) task. This hybrid psychological refractory period (PRP) paradigm created a functional processing bottleneck during memory consolidation in the visual-vocal short-term memory task. To examine how this cognitive bottleneck influences balance control, 48 participants per experiment stood quietly on a force plate, and balance control was quantified as moment variability (mN$\cdot$m) in 100 ms sliding windows. We varied the stimulus-onset asynchrony (SOA: 100 vs. 1,000 ms) between the targets (Experiment 1) and task load (report vs. ignore the visual object; Experiment 2). As expected, auditory-manual RTs increased at short SOA, showing dual-task interference that persisted in ignore trials, consistent with task-set inertia. Force-plate data were analysed using cluster permutation analysis to identify time-specific effects. Participants were less likely to adjust balance during cognitive task processing and more likely after task completion, independent of the presence of a cognitive bottleneck. These findings suggest that balance control flexibly delays or advances balance adjustments based on cognitive demands, thereby reducing cognitive-motor interference.

**Public significance statement:**

This study shows that when people are performing demanding cognitive tasks, such as remembering information while responding to auditory signals, balance adjustments can be temporarily reduced or altered, particularly when the cognitive tasks are difficult. These findings highlight the interaction of cognitive tasks and balance and specifically provide insights into how cognitive processes influence stability during standing. Our understanding of the mechanisms linking cognition and balance may guide future studies on how such interactions change with age or cognitive impairment.

## Introduction

Balance control in upright stance involves complex sensorimotor processes resulting from an interplay between the body’s displacement due to gravity and counteracting muscle torques generated around the ankle and hip joints (Winter, 1995). Traditional approaches of active balance control model standing as a continuous closed-loop process in which the motor control system continuously regulates muscle activity, incorporating sensory feedback from visual, vestibular, and somatosensory systems (Peterka, 2002).

Extending these closed-loop theories, the concept of intermittent control^1^ suggests that during standing, the current body state is continuously monitored while control impulses occur periodically in a predictive open-loop fashion to counteract balance disturbances and to maintain stability (e.g., Bottaro et al., 2008; Gawthrop et al., 2011). This view represents one possible account of balance control alongside other continuous or hybrid control models of upright stance (e.g., Ivanenko & Gurfinkel, 2018; Peterka, 2002; Winter, 1995), yet all assume the presence of corrective adjustments that are indispensable for maintaining balance (Loram et al., 2011).

Traditionally, balance control and cognitive control were studied as separate domains (D. A. Rosenbaum, 2005), but it is increasingly recognised that these systems interact and influence each other (e.g., Johannsen et al., 2023; Melzer et al., 2001; Patterson et al., 2013; D. Rosenbaum et al., 2017; Smith et al., 2019; VanderVelde et al., 2005; Ward et al., 2022). The interference between balance control and cognitive control during upright standing has increasingly been investigated.

On the one hand, studies found that when the demand of balance or cognitive tasks increases (e.g., when standing on both legs vs. one leg, or when conducting one cognitive task vs. two cognitive tasks), the interference between balance and cognitive tasks is stronger, resulting in a less stable stance or decreased performance in cognitive tasks (e.g., Patterson et al., 2013; VanderVelde et al., 2005). On the other hand, studies found that some cognitive tasks (e.g., visual tasks involving gaze shifts, Bonnet & Baudry, 2016) can even facilitate balance control. Based on the finding that relatively easy cognitive tasks can even enhance balance control while more demanding tasks impair balance control, it has been proposed that the influence of cognitive tasks on balance control depends on the specific cognitive demands (Lacour et al., 2008). Generally, effects of cognitive demands on balance control are more consistent for younger adults, whereas the results show inconsistencies for older adults (e.g., Boisgontier et al., 2013; Kang & Lipsitz, 2010; Melzer et al., 2001; Redfern et al., 2009, 2018).

One limitation of cognitive-motor interference research might be that traditional methods of analysing balance control aggregate data over long periods of time. This aggregation renders it difficult to isolate the influence of specific cognitive processes on balance adjustments (Fraizer & Mitra, 2008).

To overcome the limitation of aggregated data, a study by Johannsen et al. (2023) used an event-related approach to investigate how single cognitive processes affect concurrent balance control during standing. For the event-related approach, they continuously measured balance-control parameters and calculated means and standard deviations of ground reaction moments in time bins of 150 ms before and after both stimulus onset and response execution in each trial. This approach can be seen as analogous to event-related potentials (ERPs) in EEG studies (Luck, 2005). Specifically, Johannsen et al. (2023) used the Simon task to examine if the resolution of conflict at the response level affected balance control.

The Simon task examines how irrelevant spatial information interferes with response selection (Simon & Rudell, 1967; see Hommel, 2011, for a review). In the study by Johannsen et al. (2023) participants responded to a stimulus based on its colour but the spatial location of the stimulus could be congruent or incongruent with the required response location (a left or right manual response). The congruency effect is characterized by worse performance in incongruent compared to congruent trials. Johannsen et al. (2023) found the expected congruency effect on reaction times (RTs) and error rates of the cognitive task. When investigating the effects of spatial congruency on parameters of balance control, they found that the mediolateral (left-right) moment variability within 150 ms before the onset of the manual response was smaller in incongruent trials compared to congruent trials.

Johannsen et al. (2023) argued that incongruent trials create a spatial response conflict that delays response selection and initiation in the Simon task, and this delay might spread to the balance domain, suppressing balance control adjustments. Specifically, Johannsen et al. (2023) suggested an overlap of capacity-limited cognitive processes (required for resolution of response conflict and selection of a manual response) and processes involved in balance adjustments (intermittent control impulses). The reduction of moment variability could thus suggest a “micro-bottleneck” which omits or delays intermittent control impulses that would have been triggered close in time to the response-selection process.

In contrast to the Simon task, which investigates spatial conflicts in response selection processes, the dual-task paradigm involves performing two cognitive tasks to investigate limitations of central processing stages which can create a (cognitive) bottleneck. Specifically, in the psychological refractory period (PRP) paradigm (see Pashler, 1994; for a recent review see Koch et al., 2018) participants are presented with two stimuli in rapid succession and are required to respond to both stimuli as accurately and as quickly as possible. The interval between the two stimuli is the stimulus-onset asynchrony (SOA). As the SOA decreases, there is typically a delay in responding to the second stimulus. This response delay has been termed PRP-effect.

Traditional explanations of the PRP-effect posit a central processing bottleneck that limits parallel processing of multiple tasks at the level of response-selection (Pashler, 1994, 1998, for a review). However, subsequent empirical evidence showed that interference also occurs when encoding and retrieving information in short-term memory (see Jolicoeur et al., 2002, for a review). For example, Jolicoeur and Dell’Acqua (1998) used a dual task consisting of a visual short-term memory task followed by a speeded auditory-manual two-choice task. Their results suggested that the encoding and consolidation of the visual-vocal short-term memory task interfered with response selection in the second task (Koch et al., 2003; Koch & Jolicoeur, 2007; see also Koch & Prinz, 2002).

Based on a study by Jolicoeur and Dell’Acqua (1998); Koch and Rumiati (2006) investigated the influence of processing a briefly presented and subsequently masked visual object (e.g., a mug) that had to be reported at the end of the trial (visual-vocal short-term memory task) on performance in an unrelated speeded two-choice tone-discrimination task (auditory-manual RT task). For the visual-vocal short-term memory task, participants had to report the orientation of the object at the end of the trial. The verbal report had to be given after they completed the auditory-manual RT task by saying “left” or “right” (in German “links” and “rechts”). This task requires an encoding and memory-consolidation process. The target stimulus in the auditory-manual RT task was a low- or high-pitched tone, which was presented after the visual object with a variable and unpredictable SOA. Participants had to respond as fast and accurately as possible to the tone by manually pressing a key with one of the index fingers. Using these dual-task requirements, Koch and Rumiati (2006) found that RTs were increased in trials with short SOA compared to long SOA. This was interpreted as a functional processing bottleneck during memory-consolidation processes in the visual-vocal short-term memory task, which interfered with response selection in the auditory-manual RT task due to limited cognitive resources. Hence, at short SOA consolidating the representation of the visual object into short-term memory might not be completed when a response for the auditory target has to be selected, delaying response selection.^2^

Furthermore, Koch and Rumiati (2006) manipulated whether participants had to report or ignore the (always presented) visual object on a trial-by-trial basis in their second experiment. The results showed SOA effects (increased RTs for short compared to long SOA trials) in both report and ignore trials, but the SOA effect was much smaller in ignore (i.e., single task) trials. This residual SOA effect in ignore trials suggests that participants processed the visual object to some degree even if it was not required by the task, but the SOA effect in report trials was substantially larger indicating the time required for memory consolidation.

In the present study, we used the dual-task approach by Koch and Rumiati (2006) to assess whether the resulting dual-task effects would carry over to balance control during upright bipedal stance as less cognitive capacity is available for balance adjustments, leading to an interference effect between the cognitive task and motor control. The specific type of dual task is particularly well suited to our study, as memory-consolidation processes and their interference with response-selection processes do not themselves involve any overt motor components. This allowed us to analyse the influence of cognitive processes on balance control before the manual response to the auditory-manual RT task without possible confounding motor actions like the preparation of a manual response. Balance control, although central to our research question, was not treated as an additional instructed task but as a continuously operating motor control process that provides the context within which cognitive dual tasking occurs. We analysed balance control in an event-related way by continuously recording body sway with a force plate and aggregating data using a running integration window within a given trial. Importantly, this study takes an exploratory approach to investigating how cognitive dual-task demands might influence balance control in the range of sub-second time periods.

## Overview of experiments

In the current experiments, the influence of cognitive dual-task demands on balance control during standing was investigated in two experiments. In Experiment 1, SOA (100 vs. 1,000 ms) varied randomly from trial to trial and participants always had to report the visual object at the end of each trial. In Experiment 2, in addition to the SOA variation, we randomly implemented a single-task condition in which the visual object was presented but could be ignored (task load: report vs. ignore) to test whether effects of SOA would persist in ignore trials and if the absence of a cognitive bottleneck would have an influence on balance control.

## Experiment 1

In Experiment 1, we adapted the dual-task paradigm by Koch and Rumiati (2006) and asked participants to perform two tasks while standing on a force plate. This allowed us to continuously measure sway parameters and analyse moment^3^ variability on the force plate in each trial using an event-related approach similar to that of Johannsen et al. (2023).

Regarding the cognitive task, we expected that performance in the auditory-manual RT task would decrease with short compared to long SOA. To assess potential interference or facilitation effects on balance control, we analysed the force-plate data in an exploratory way. For this, we considered multiple time windows over the course of each trial and analysed if the SOA variation had any systematic effects on moment variability.

### Method

#### Participants

An a priori sample size calculation was performed in G*Power (version 3.1.9.7, Faul et al., 2007), assuming a small to medium Cohen’s *f* effect size of 0.18 (based on data from a pilot study), an $\alpha$-error probability of 0.05 and a power of 0.95 for repeated measures ANOVAs, resulting in a sample size of 44 participants. We decided to collect 48 participants to ensure proper counterbalancing.

Data were collected in two laboratories, with basically identical setup, in Aachen and Freiburg (both Germany).^4^ Forty-eight participants (36 female, 11 male, and one diverse, mean age = 20.9, four left-handed) took part in the experiment and received course credits. All participants reported normal or corrected-to-normal vision and hearing acuity, and no balance problems. Written informed consent was given and data were handled in line with the German national data protection law. Ethical approval was granted by the Ethics Committee of RWTH Aachen University (29.10.2023/2023_17_FB7_RWTH AACHEN) prior to the experiment and the experiment complied with the Declaration of Helsinki.

#### Apparatus, stimuli, and tasks

The experiment was conducted in a dimly lit, quiet room. A 31.5” LCD monitor with a refresh rate of 144 Hz and a resolution of $3840\times 2160$ pixels was used for instructions including a preview of the visual stimuli that would be used later, and for presenting the stimuli of the visual-vocal short-term memory task. The monitor was placed in front of the participants at a distance of about 85 cm and a height of 130 cm. Headphones (Superlux HMD-660) presented the target of the auditory-manual RT task binaurally. The input device for the auditory-manual RT task was a game controller (Rii PC controller USB gamepad; dimensions: 16 $\times$ 11.5 7 cm; weight: 161 g). Participants held the game controller with both hands in front of their upper body. They were instructed to hold the controller relaxed and rather close to the body, with the elbows close to both sides of the body and the arms slightly bent upwards. Participants’ vocal responses were coded using a keyboard by the experimenter, who was in the same room but separated by a screen wall.

Body sway was recorded using a multi-component force plate type Kistler 9260AA with a temporal resolution of 1,000 Hz. It uses piezoelectric 3-component force sensors to record the forces and moments exerted on the plate from above in the three spatial directions. The experiment was run on a computer using PsychoPy^®^ (version 2023.2.3, Peirce et al., 2022). The sway measurement was performed on a second computer using the BioWare^®^ software (version 5.3.0.7, Kistler Group, 2012). The computer running PsychoPy^®^ communicated via a parallel port with the force-plate computer. Unique signals were sent at several events (fixation-cross onset, target onset for the auditory-manual RT task and for the visual-vocal short-term memory task, and response onset for the auditory-manual RT task) in each trial for later synchronisation and characterisation of behavioural and force-plate data (see Hartmann et al., 2025 for more information about this setup).

**Fig. 1:**
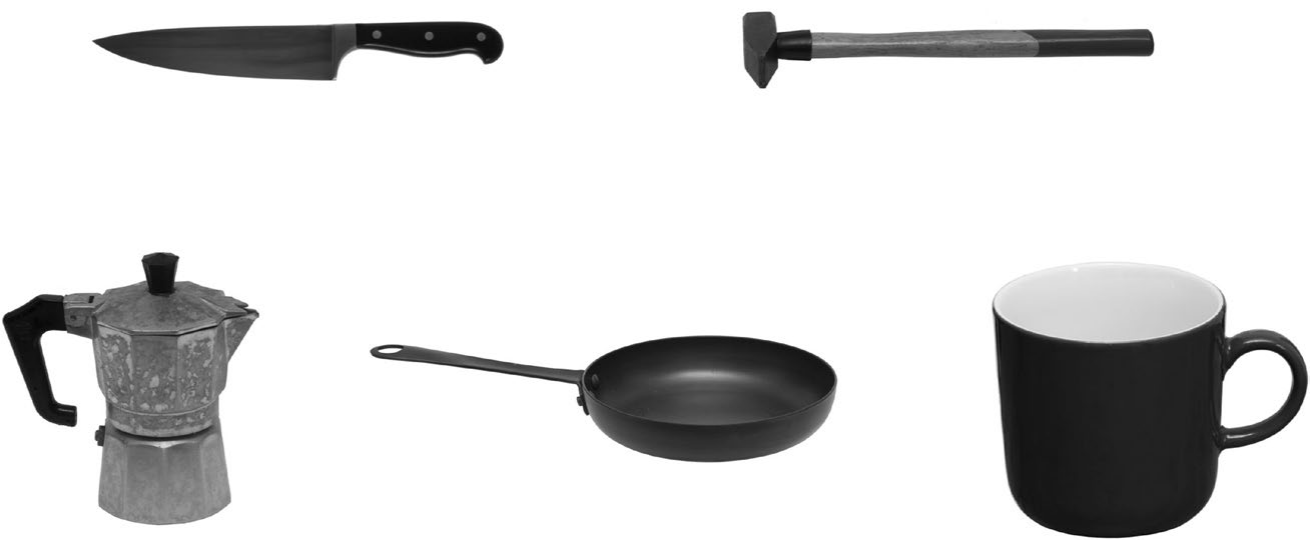
Objects used as visual target stimuli. Each of the five stimuli was presented in left or right orientation. Stimuli were presented centrally in colour with low saturation in front of a white squared background which filled part of the screen

The visual target stimuli for a visual-vocal short-term memory task were photographs of five everyday objects on a white background inspired by Koch and Rumiati (2006). These objects were presented either straight or mirrored so that they could be grasped either on the left or right side (e.g., a pan with the handle on the left or on the right; see Fig. 1 for all five objects). Responses to the visual objects were the words “left” or “right” (in German: “links” or “rechts”), which had to be given vocally after the speeded response to the tone in the auditory-manual RT task. Auditory target stimuli for an auditory-manual RT task were tones generated by PsychoPy^®^ with a low frequency (200 Hz) or a high frequency (600 Hz). Participants were instructed to respond with a left or right key press to a low- or high-pitched tone with the index finger on the game controller (the assignment was counterbalanced over participants).

#### Procedure

**Fig. 2:**
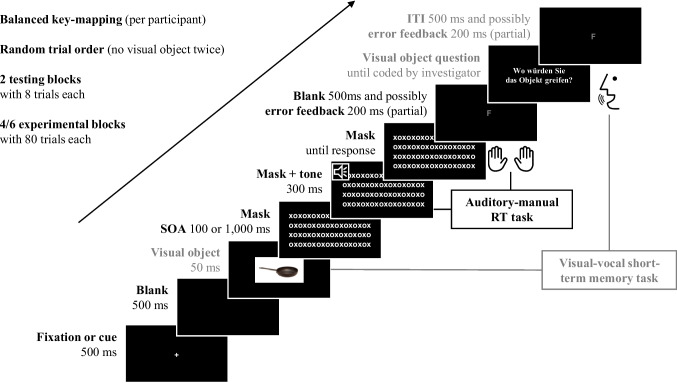
Trial sequence and experiment information. Each trial consisted of a visual short-term memory task with a delayed verbal response (grey font colour) and an auditory-manual reaction time task. Balanced key-mapping refers to the mapping of the auditory target stimulus (200 or 600 Hz) and manual response (left or right key press). In Experiment 2, the same trial sequence was used, preceded by a visual cue indicating whether the visual object had to be reported or ignored

Participants stood on the force plate with feet hip-width apart in upright bipedal stance without shoes. The experiment consisted of two practice blocks of eight trials each and four main blocks of 80 trials each. No explicit rest breaks were scheduled, but short self-paced pauses occurred between blocks while data were saved and the next block was initialized. Participants did not report any signs of fatigue or discomfort. All stimuli were presented against black background. Each trial started with a fixation cross which was presented for 500 ms (see Fig. 2 for a detailed trial structure). This was followed by a blank screen for 500 ms before the visual target was presented for 50 ms. The visual object was then masked with a $16\times 8$ array of “x” and “o” letters. After a varying SOA (100 vs. 1,000 ms after visual target onset), a tone (low: 200 vs. high: 600 Hz for the auditory-manual RT task) was played through headphones for 300 ms. The visual mask remained on the screen until participants manually responded to the tone by pressing the left or right key. The response was then followed by a blank screen for 500 ms, with possible error feedback (letter “F” in red colour) presented in the first 200 ms. Then, a prompt appeared on the screen asking participants where to grasp the visual object (in German: “Wo würden Sie das Objekt greifen?”), focusing on accuracy not speed (which we did not analyse). This message remained on the screen until the experimenter coded the participants’ vocal response, followed by a blank screen of 500 ms, with a possible error feedback (letter “F” in red colour) in the first 200 ms. The next trial always started after a blank screen of 500 ms.

Each combination of visual target orientation (left and right), auditory tone (low and high; requiring left and right responses), and SOA (100 and 1,000 ms) were presented 10 times per block in quasi-random order. All five objects were presented equally often in quasi-random order with the exception that the same visual object (regardless of its orientation) was not shown in two consecutive trials. The S–R mapping of the auditory-manual RT task was counterbalanced across participants (left key for high tone vs. left key for low tone).

At the end of the experiment, participants completed a follow-up questionnaire asking about their athleticism and the difficulty of the experiment, and a debriefing. The whole experiment took about 45 minutes.

#### Design

The independent variable was SOA (100 vs. 1,000 ms). The dependent measure for the visual-vocal short-term memory task was error rates. For the auditory-manual RT task, we measured RTs and error rates. To analyse the data of the cognitive dual task, we conducted separate paired samples *t*-tests for each task, with a focus on the auditory-manual RT task.

For the behavioural analyses, we tested the normality of residuals using the Shapiro–Wilk test (Shapiro & Wilk, 1965). Although slight deviations from normality were observed, parametric tests such as the paired-samples *t*-test and repeated-measures ANOVA are robust to moderate violations of this assumption, particularly with the present sample size of 48 participants per experiment (see Blanca Mena et al., 2017; Schmider et al., 2010). The main analyses of the force-plate data relied on cluster-based permutation tests, which are nonparametric and not subject to distributional assumptions (Maris & Oostenveld, 2007).

#### Force-plate data

Following, we will discuss the force-plate data preparation and analysis. The force-plate data were filtered using a 4$^{th}$ order dual-pass *Butterworth* low-pass filter with a cut-off frequency of 10 Hz (adapted from Johannsen et al., 2023). This approach is commonly used to smoothen force-plate time-series data, helping to eliminate high-frequency signals, artifacts, or byproducts that are not directly related to supraspinal balance control, such as electromagnetic interference or peripheral and spinal responses (e.g., muscle twitches or spinal reflexes, Christensen et al., 2000). By setting the cut-off frequency at 10 Hz, the shortest cycle duration of oscillatory signals was 100 ms, matching the duration of our integration windows. Consequently, this analysis focuses on supraspinal mechanisms of balance control, including long-latency (transcortical) reflexes and more complex balance-related adjustments. Data were processed using the R package forceplate (Hartmann et al., 2025; Hartmann & Koger, 2024). With the package, we segmented the continuous force-plate data into single trials and aligned data at the fixation-cross onset.^5^ The events of interest within each trial are the fixation-cross onset, visual target onset, auditory target onset, and onset of the manual response. We decided to always align data at fixation-cross (or cue) onset, as we discovered that moment variability continuously decreased over the course of a trial in our experiments. Aligning our force-plate data at the manual response would result in systematically different absolute time points after the beginning of a trial as the manual response times differed between experimental manipulations, making conclusions about the manipulations on balance control difficult. We decided to not only analyse moment variability at specific events but throughout the entire trial. For this, we used a running integration window to process force-plate data by computing a statistic (here, standard deviation) over a sliding window that moves across a time series. In each time step (here, 1 ms), only data points within a defined time window (here, 100 ms wide) are considered. This procedure allows us to capture the temporal dynamics of moments over the course of a trial while maintaining the temporal resolution of 1,000 Hz. Like this, we preserved local features which might be lost when only analysing non-overlapping time bins (e.g., with EEG and fMRI data, Kiviniemi et al., 2011; Parameshwaran & Thiagarajan, 2023). Due to the large number of integration windows in each trial, we decided to use cluster permutation analysis for a more exploratory purpose (Maris & Oostenveld, 2007; for a general overview see Everitt et al., 2011).

Cluster permutation analysis is a statistical technique used primarily in neuroimaging (e.g., Takacs et al., 2020; Wang et al., 2024) and for time-series data (e.g., Miklashevsky et al., 2022 for grip force; Gresch et al., 2024 for eye movements) to identify clusters of data points that differ significantly from each other across different experimental conditions while controlling for multiple comparisons. This analysis is particularly useful when dealing with complex data structures where dependencies exist between adjacent data points. This method involves randomly shuffling data labels to create a distribution of cluster masses under the null hypothesis, allowing researchers to determine the likelihood of observed clusters occurring by chance. The process begins by computing *t*-statistics for each data point across conditions. Data labels are then randomly shuffled multiple times, and for each permutation, the *t*-statistics are recalculated. Clusters of adjacent data points that exceed a predefined significance threshold are identified, and their “mass” (sum of *t*-statistics within the cluster) is recorded. This generates a null distribution of cluster masses. The observed cluster masses from the original data are compared against this null distribution to determine their statistical significance. If an observed cluster’s mass is larger than 95% or 99% of the mass of clusters in the null distribution, it is deemed statistically significant (Maris & Oostenveld, 2007). One of the key advantages of cluster permutation analysis is its ability to account for dependencies across data points. Unlike traditional methods that analyse time-series data independently, this method considers entire data profiles, reducing the risk of overlooking significant effects that span multiple time points. Additionally, it avoids the issue of inflated Type I error rates that arise from conducting multiple comparisons, offering a more robust solution for identifying true effects in the data. While the cluster permutation analysis effectively controls for multiple comparisons and accounts for temporal dependencies, it may show some imprecision in defining the exact start and end points of significant clusters and can slightly overestimate their duration in noisy data (Sassenhagen & Draschkow, 2019).

The dependent measure of balance control was the variability of force moments (also referred to as moment variability in millinewton metres: mN$\cdot$m). The moment variability within an integration window reflects the amount of neuromuscular activity devoted to maintain balance, with higher moment variability indicating a greater likelihood of balance adjustments being made within that time frame (Gawthrop et al., 2011; Johannsen et al., 2023).

We chose to use moment variability instead of center of pressure (CoP) data because moment variability directly reflects the neuromuscular effort involved in balance adjustments (Gawthrop et al., 2011). Moreover, the standard deviation of force moments has been successfully applied in a similar experimental design, making it an appropriate and comparative measure in the range of milliseconds for assessing balance control under different cognitive task conditions (Johannsen et al., 2023).

We analysed moment variability combined for mediolateral (left-right) and anterior-posterior (front-back) directions (by averaging) in integration windows of 100 ms from 500 ms before fixation-cross onset to 3,000 ms after fixation-cross onset. For cluster permutation analysis, we used the R package “permuco” (Frossard & Renaud, 2021) and permuted the data 2,000 times. All significance tests were performed at an alpha level of 0.05.

#### Transparency and openness

In accordance with the Journal Article Reporting Standards (JARS, Appelbaum et al., 2018), we report how we determined our sample size, all data exclusions, all manipulations, and all measures in the study. All data, analysis code, and research materials are available.^6^ The data were analyzed using R (version 4.3.0, R Core Team, 2023). This study was not preregistered.

### Results

Overall, 1.6% of trials were excluded because of deviating more than 3 *SD* from the mean RT per participant. For the analysis of error rates in the visual-vocal short-term memory task, all trials with errors in the auditory-manual RT task and trials following an error were excluded (3.9%). For the analysis of error rates in the auditory-manual RT task, all trials with errors in the visual-vocal short-term memory task and trials following an error were excluded (4.1%). For the RT analysis in the auditory-manual RT task, all trials with errors in the auditory-manual RT task or visual-vocal short-term memory task and trials following an error were excluded (7.7%). Finally, all remaining first trials in each block were excluded as the experimenter started the blocks for the participants. For the analysis of force-plate data, we excluded the same trials as for the RTs in the auditory-manual RT task.

Results are presented separately for the cognitive dual task (error rates for the visual-vocal short-term memory task; error rates and RTs for the auditory-manual RT task) and for the force-plate data (moment variability for each millisecond in running integration windows of 100 ms over the course of each trial).

#### Cognitive dual task

The SOA manipulation did not significantly impact error rates in the visual-vocal short-term memory task, $t(47) = -0.59$, $p = .559$, Cohen’s *d* = 0.08 (1.9% vs 2.1% for short vs. long SOA).

**Fig. 3:**
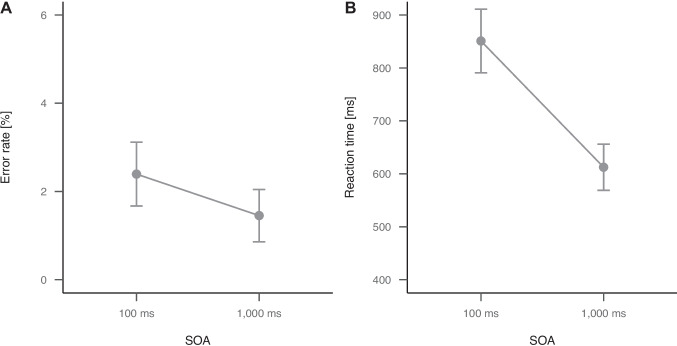
Plots of the error rates (A) and RTs (B) of the auditory-manual RT task in Experiment 1 as a function of SOA. Error bars indicate 95% confidence intervals

The analysis of error rates in the auditory-manual RT task revealed a significant main effect of SOA, $t(47) = 3.82$, $p < .001$, Cohen’s *d* = 0.41, with more errors in short SOA trials (2.4%) compared to long SOA trials (1.5%). All effects are shown in panel A of Fig. 3.

The analysis of RTs in the auditory-manual RT task revealed a significant main effect of SOA, $t(47) = 17.44$, $p < .001$, Cohen’s *d* = 1.32, with longer RTs in short SOA trials (851 ms) compared to long SOA trials (613 ms) and thus a dual-task effect of 238 ms.^7^ All effects are shown in panel B of Fig. 3.

#### Force-plate data

**Fig. 4:**
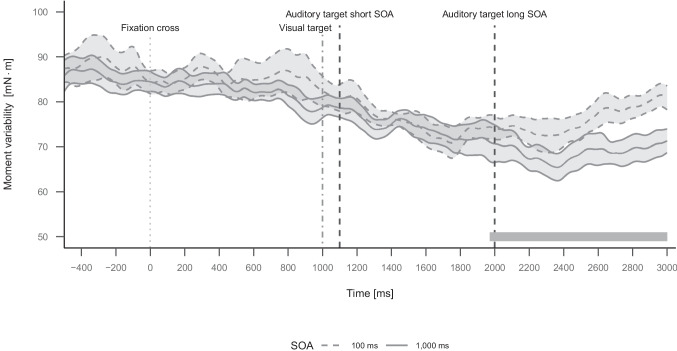
Plot of moment variability over the course of a trial in Experiment 1 as a function of SOA. Moment variability was averaged about mediolateral (left-right) and anterior-posterior (front-back) axis. Data were aligned at fixation-cross onset. The visual target appears at 1,000 ms. The auditory target appears at 1,100 or 2,000 ms depending on the SOA condition. Events are indicated by vertical lines. The grey area displayed above the x-axis represents a time window with a significant cluster. Error ribbons indicate 95 % confidence intervals

The cluster permutation analysis identified a cluster of SOA from 1,971 to 3,000 ms after fixation-cross onset, *p* =$\leq .001$, and a cluster mass of 18,529.29, indicating significantly more moment variability for short compared to long SOA. The cluster of SOA is shown in Fig. 4.

### Discussion

For the auditory-manual RT task, our results replicated the findings of Koch and Rumiati (2006). As expected, participants responded more slowly in the auditory-manual RT task for short than long SOA. This finding is in line with the assumption of a bottleneck between memory-consolidation processes for the visual object, which interfered with the response-selection process in the auditory-manual RT task. The masking of the visual object required immediate encoding, thus memory performance was highly accurate and independent of SOA but it produced a substantial dual-task interference effect on performance in the auditory-manual RT task.

In the force-plate data, we did not find a significant influence of the SOA manipulation on moment variability for the time during which the memory-consolidation bottleneck occurred (after auditory target onset in trials with short SOA from around 1,100 to 1,400 ms). Interestingly, we found significantly more moment variability towards the end of the analysis window in trials with short SOA compared to trials with long SOA. In trials with long SOA, participants still had to prepare for or process the auditory target, thus focus their attention on the auditory-manual RT task. In contrast, in trials with short SOA, participants had already completed the manual response to the auditory target. Similar to the results by Johannsen et al. (2023), the manual response selection process in our dual-task paradigm might spread to the balance domain and thus result in less balance adjustments being executed prior to the manual response as selecting a manual response could impair the quality of balance adjustments. Finally, the likelihood of balance adjustments increases again after the manual response, showing that balance adjustments are more likely again after task completion (see Bohlke et al., 2021, who looked at post-task balance).

Overall, moment variability and thus the likelihood of balance adjustments was higher around the fixation-cross until the visual target and lower before the manual response execution (around 1,750 ms in trials with short SOA and around 2,400 ms in trials with long SOA). We suggest that this is the case because participants delay or advance balance adjustments to minimize cognitive-motor interference.

Importantly, one critical limitation of Experiment 1 is based on the complex nature of force-plate data where dependencies exist between adjacent data points. Thus, analysing the effect of SOA on moment variability after the auditory target onset is difficult, as the auditory target occurred earlier in trials with short compared to long SOA. To enable a comparison of moment variability at more similar time points under varying task load conditions, we implemented report and ignore trials as Koch and Rumiati (2006) did in Experiment 2, resulting in dual and single-task like conditions. Hence, we can compare report (dual-task) and ignore (single-task) trials at identical absolute points in time, while they differ in their cognitive demands (task load), thus employing another operational definition of dual-task costs (i.e., dual vs. single).

## Experiment 2

In Experiment 2, we additionally manipulated whether participants had to report or ignore the visual-vocal short-term memory task. The levels of task load (report vs. ignore) were randomly varied within blocks. We hypothesized that performance in the auditory-manual RT task would decrease when participants had to report the visual object (dual task) compared to when they had to ignore it (single task). Furthermore, we expected larger dual-task costs in trials with short compared to long SOA. Again, we approached the force-plate data in an exploratory way and analysed if the factors SOA and task load had any systematic effect on moment variability over the course of each trial.

### Method

#### Participants

Forty-eight new participants (42 female and 6 male, mean age = 21.2, four left-handed) successfully took part in Experiment 2 in a lab in Aachen (Germany) and received course credits.

#### Apparatus, stimuli, and procedure

Apparatus and stimuli were the same as in Experiment 1. We increased the trial numbers by adding two blocks resulting in 480 trials in total. In addition, each trial started with a 500 ms cue (instead of a fixation cross) which informed participants whether they should ignore the visual object or if they should pay attention to the always presented visual object (German: “Objekt ignorieren” vs. “Objekt beachten”; English: “ignore object” vs. “attend object”). When participants were instructed to ignore the visual object, the trial ended after the manual response to the auditory target. If participants were instructed to pay attention to the visual object, the trial continued like all trials in Experiment 1. The whole experiment took about 1 hour.

#### Design

Experiment 2 used a $2\times 2$ repeated measures design. Independent variables were SOA (100 vs. 1,000 ms) and task load (report vs. ignore). For the analysis of the error rates of the visual-vocal short-term memory task, we computed a paired samples *t*-test, as participants only responded to the visual object in report trials which left us with the factor SOA. For the analysis of the auditory-manual RT task, we computed $2\times 2$ repeated measures ANOVAs with the factors SOA and task load separately for RTs and error rates. The dependent measure of balance control was moment variability in integration windows of 100 ms over the course of each trial. Like in Experiment 1, we analysed data with a cluster permutation analysis. Four cluster permutation analysis were run in total to analyse the effects of SOA and task load on moment variability over the course of a trial. To compare the influence of the SOA manipulation (100 vs. 1,000 ms), we ran the analysis separately for report and ignore trials. To compare the influence of the task load manipulation (report vs. ignore), we ran the analysis separately for short and long SOA trials. It should be noted that when participants could ignore the visual object, trials with short SOA actually ended after the manual response around 1,800 ms.

### Results

Exclusion criteria were equal to Experiment 1. Overall, 1.7% of trials were excluded because of deviating more than 3 *SD* from the mean RT per participant. For the analysis of error rates in the visual-vocal short-term memory task, all trials with errors in the auditory-manual RT task and trials following an error were excluded (4.2%). For the analysis of error rates in the auditory-manual RT task, all trials with errors in the visual-vocal short-term memory task and trials following an error were excluded (5.2%). For the RT analysis in the auditory-manual RT task, all trials with errors in the auditory-manual RT task or visual-vocal short-term memory task and trials following an error were excluded (8.8%). Finally, all remaining first trials in each block were excluded as the experimenter started the blocks for the participants. The force-plate data were filtered and processed like in Experiment 1. For the analysis of force-plate data, we excluded the same trials as for the RT-analysis in the auditory-manual RT task.

#### Cognitive dual task

The SOA manipulation did not significantly impact error rates in the visual-vocal short-term memory task, $t(47) = -0.83$, $p = .412$, Cohen’s *d* = 0.08 (5.0% vs 5.3% for short vs. long SOA).

**Fig. 5:**
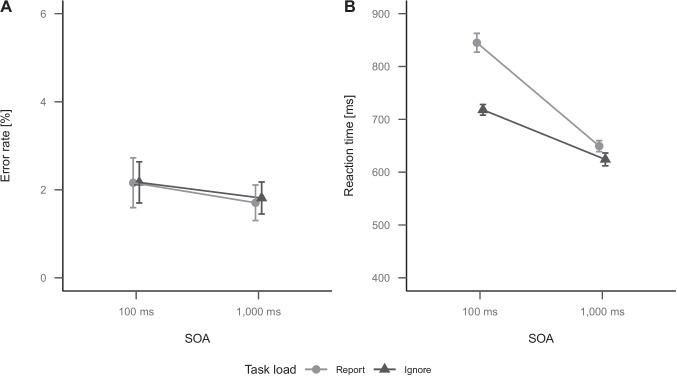
Plots of the error rates (A) and RTs (B) of the auditory-manual RT task in Experiment 2 as a function of SOA and task load. Error bars indicate 95% confidence intervals

The analysis of error rates in the auditory-manual RT task revealed a significant main effect of SOA, $F(1, 47) = 5.98$, $p = .018$, $\eta ^2_p$ = 0.11, indicating higher error rates for short SOA (2.2%) than for long SOA (1.8%). All other effects were not significant, *F* $\le$ 0.07 and *p* $\ge$ .796. All effects are shown in panel A of Fig. 5.

The analysis of RTs in the auditory-manual RT task revealed a significant main effect of SOA, $F(1, 47) = 175.72$, $p < .001$, $\eta ^2_p$ = 0.79, indicating longer RTs for short SOA (782 ms) than for long SOA (637 ms). The main effect of task load was also significant, $F(1, 47) = 90.05$, $p < .001$, $\eta ^2_p$ = 0.66, indicating longer RTs for report (747 ms) compared to ignore trials (671 ms) and thus 76 ms dual-task costs. The interaction of SOA and task load was significant, $F(1, 47) = 81.13$, $p < .001$, $\eta ^2_p$ = 0.63. The SOA effect was clearly larger for report trials (196 ms) but still present for ignore trials (94 ms).^8^ All effects are shown in panel B of Fig. 5.

#### Force-plate data

**Fig. 6:**
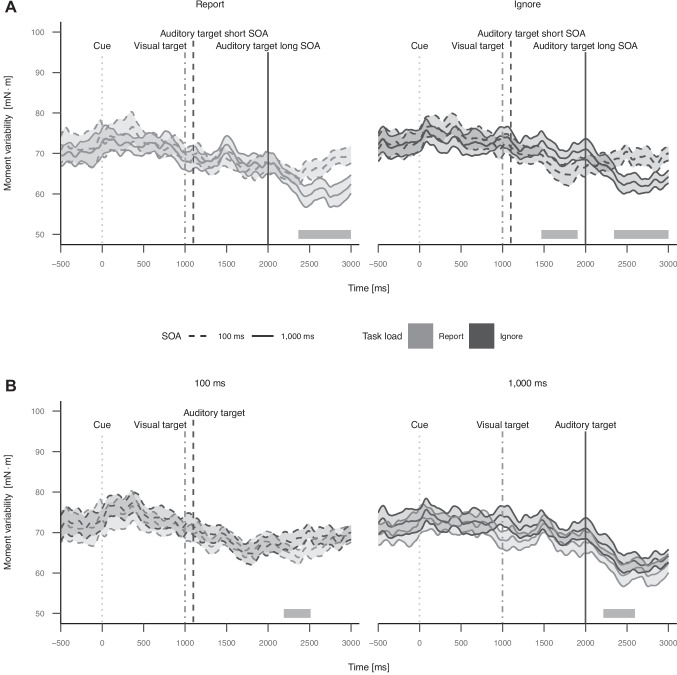
Plots of moment variability over the course of a trial in Experiment 2 to show effects of SOA (A) and task load (B). Data were aligned at cue onset. The visual target appears at 1,000 ms. The auditory target appears at 1,100 or 2,000 ms depending on the SOA condition. Events are indicated by vertical lines. The grey areas displayed above the x-axis represent time windows with significant clusters. Error ribbons indicate 95 % confidence intervals

The cluster permutation analysis for report trials identified a cluster of SOA from 2,367 to 3,000 ms after cue onset, *p* $\leq$ .001, and a cluster mass of 10,861.38, indicating significantly more moment variability for short compared to long SOA, thus replicating the finding from Experiment 1. The cluster of SOA for report trials is shown on the left side in panel A of Fig. 6.

The cluster permutation analysis for ignore trials identified two clusters of SOA. The first one from 1,468 to 1,905 ms after cue onset, *p* = .013, and a cluster mass of 3,009.68, indicating significantly less moment variability for short compared to long SOA. This effect is in the opposite direction compared to Experiment 1 but the cluster appears around 500 ms earlier compared to the significant cluster in Experiment 1. It should be mentioned that in trials with short SOA, the auditory target occurs before this cluster while in trials with long SOA, the auditory target is yet to come. The second cluster ranged from 2,314 to 3,000 ms after cue onset, *p* $\leq$ .001, and a cluster mass of 10,043.70, indicating significantly more moment variability for short compared to long SOA, thus replicating the finding from Experiment 1. The clusters of SOA for ignore trials are shown on the right side in panel A of Fig. 6.

The theoretically most interesting cluster permutation analysis for trials with a short SOA identified a cluster of task load from 2,193 to 2,514 ms after cue onset, *p* = .034, and a cluster mass of 2,170.04, indicating significantly less moment variability for report compared to ignore trials. The cluster of task load for short SOA trials is shown on the left side in panel B of Fig. 6.

The cluster permutation analysis for trials with a long SOA identified a cluster of task load from 2,216 to 2,597 ms after cue onset, *p* = .045, and a cluster mass of 1,926.91, indicating significantly less moment variability for report compared to ignore trials. The cluster of task load for long SOA trials is shown on the right side in panel B of Fig. 6.

### Discussion

In Experiment 2, we aimed to further investigate the influence of cognitive dual-task demands on balance control during standing. In the auditory-manual RT task, we successfully replicated Experiment 1 as well as the findings of Koch and Rumiati (2006). Regarding task load, we observed that auditory-manual RT task performance was better in trials where participants were instructed to ignore the visual object compared to those where they had to report it, giving another measure of dual-task costs. Moreover, in ignore trials, the SOA effect was smaller but did not disappear. This suggests that when participants could ignore the visual object, they still residually processed the object at least in some trials, similar to what was found by Koch and Rumiati (2006).

In the force-plate data, like in Experiment 1, we did not find a significant influence of the SOA manipulation on moment variability for the time during which the assumed memory-consolidation bottleneck occurred (after auditory target onset in report trials with short SOA from around 1,100 to 1,400 ms). As in Experiment 1, we found significantly more moment variability towards the end of the analysis window for trials with short SOA compared to trials with long SOA in Experiment 2. This pattern was independent of the task load manipulation (report vs. ignore). Additionally, we found an earlier cluster with significantly less moment variability for short compared to long SOA and thus in the opposite direction in ignore trials only. This effect occurred just before the manual response in trials with short SOA and did not occur in report trials, where the memory consolidation of the visual-vocal short-term memory task overlapped with the response selection process of the auditory-manual RT task. This reduction in moment variability might reflect inhibition of the irrelevant visual target during response-selection processes in these ignore trials with short SOA, which could carry over to balance control and transiently reduce the likelihood of executing balance adjustments.

Overall, as in Experiment 1, moment variability and thus the likelihood of balance adjustments was higher around the cue until the visual target and lower before the manual response execution (which is at different time points depending on the SOA condition), supporting the idea that participants delay or advance balance adjustments to minimize or optimize cognitive-motor interference.

We did not find a significant influence of the task load manipulation on moment variability in short SOA trials for the time during which the assumed memory-consolidation bottleneck occurred when comparing report and ignore trials. But task load significantly affected moment variability between around 2,200 and 2,550 ms after cue onset in trials with short and long SOA, with less variability being observed in report compared to ignore trials. In trials with short SOA, the difference reflects balance adjustments being less likely while the visual object is being retrieved for vocal report in report but not in ignore trials which ended around this time. Visual information is essential for maintaining stability during upright stance, providing real-time updates on body position relative to the environment (Winter, 1995). This could be the case because retrieving the visual object might compromise the ability to utilize visual information for balance adjustments making state estimations and thus adjustments less accurate. In trials with long SOA, the retrieval of the visual object happens later. In these long SOA trials participants have to keep the visual object in short-term memory in report but not in ignore trials. This additional load might also lead to less balance adjustments due to the visual properties of the object in short-term memory which might impair the ability to utilize visual information for balance adjustments.

If the memory-consolidation bottleneck in the cognitive task interferes with balance control, there should be a difference in moment variability in the range from around 1,100 to 1,400 ms depending on the levels of SOA and task load. Specifically, we would have expected less moment variability in report compared to ignore trials with short SOA but not with long SOA. Not finding this effect could be due to residual processing of the visual object in ignore trials which might have reduced the “true” dual-task costs at short SOA. This could affect balance control because even residually processing the visual object might compromise the ability to utilize visual information for balance adjustments.

## General discussion

The present study investigated the influence of cognitive dual-task demands on balance control in the range of sub-second time periods using an event-related method. In two experiments, participants were standing on a force plate which measured sway parameters continuously while performing two cognitive tasks. This dual-task paradigm consisted of a visual-vocal short-term memory task with a delayed vocal response and a two-choice auditory-manual RT task. We manipulated the SOA (short vs. long) between the visual and auditory targets in Experiment 1. In Experiment 2, we additionally manipulated task load by instructing participants to report or ignore the object of the visual-vocal short-term memory task.

### Cognitive dual-task performance

In both experiments, the cognitive tasks revealed clear dual-task interference. Specifically, short SOA led to greater interference between tasks, particularly in the auditory-manual RT task. This interference was attributed to a memory-consolidation bottleneck, where the processing of the visual-vocal short-term memory task interfered with the response selection for the auditory-manual RT task (Jolicoeur & Dell’Acqua, 1998; Koch et al., 2003; Koch & Rumiati, 2006). Notably, this represents a PRP-like dual-task performance effect (for a review see Koch et al., 2018), but it is not caused by response selection in the first task but by the need to immediately encode the visual target and consolidate it into short-term memory.

In Experiment 2, we additionally manipulated task load by instructing participants to either report or ignore the object of the visual-vocal short-term memory task. When participants had to report the visual object compared to when they had to ignore it, performance in the auditory-manual RT task was worse for short and also for long SOA. Interestingly, even when participants were instructed to ignore the visual object, some residual degree of processing still occurred, similar to what was found by Koch and Rumiati (2006).

This data pattern suggests that a form of “task-set inertia”, in which previously activated cognitive processes continue to influence performance (for a review see Allport & Wylie, 1999; more recently see Koch & Kiesel, 2022, for a review), played a role in dual-task interference. Recently, Jung et al. (2025) examined how previously active task sets continue to influence performance even after task demands are reduced. Using a fade-out paradigm (Mayr & Liebscher, 2001) in a PRP-like dual-task setup, participants initially performed two concurrent tasks and were subsequently instructed to continue with Task 1 only. Despite clear instructions, participants showed persistent dual-task costs: RTs for Task 1 remained elevated and only gradually returned to single-task levels. The authors interpret this as evidence for a temporally extended persistence of the dual-task set, suggesting that cognitive control does not instantly adapt to reduced task demands.

### Balance control and moment variability

The most novel aspect of the present study was to explore how cognitive control demands influence balance control. The results showed no process-specific SOA and task-load effects on moment variability for the time during which the assumed memory-consolidation bottleneck likely occurred (i.e., within 500 ms after onset of the visual target). However, we found reduced moment variability in ignore trials with short compared to long SOA which might reflect inhibitory processing of the irrelevant visual target during the period of response selection in the auditory-manual RT task. This inhibition could transiently carry over to balance control, but this speculation requires further research.

Our variability measure reflects the probability of a balance adjustment occurring during a specific temporal period. Overall, we found higher moment variability around the fixation-cross/cue and the visual target and lower moment variability before the manual response selection. This consistent variability pattern suggests a specific range in which the likelihood of a balance adjustment is reduced.

A comparable transient reduction in balance adjustments has also been reported in studies on attentional orienting and postural immobility. When attention is strongly focused on demanding or salient stimuli, body sway often decreases - a phenomenon referred to as postural freezing (e.g., Lojowska et al., 2015). These studies typically examined overall sway amplitude or center of pressure velocity and, in some cases, heart-rate deceleration as physiological markers of freezing (e.g., Roelofs, 2017). Reductions in these markers are thought to reflect an adaptive stabilization that supports sensory processing or response preparation. Although our task did not include emotional stimuli, similar attentional mechanisms might have contributed to the temporary minimization of balance adjustments observed during cognitively demanding phases.

While we observe more or less moment variability, it is important to note that balance adjustments are preceded by an evaluative process that requires time, potentially leading to delayed effects in our force-plate data (Gawthrop et al., 2011). Finding reduced moment variability during the response selection independent of the memory-consolidation bottleneck suggests that balance adjustments are not solely reactive, but also influenced by the cognitive evaluation of the situation.

Our data pattern lead to the assumption that balance control exhibits sufficient flexibility to execute balance adjustments at a specific point in time potentially to facilitate performance in a concurrent cognitive task. This flexibility allows the balance control system to delay or advance balance adjustments (e.g., into the inter-trial interval, before or after the cognitive task), thereby reducing or adaptively adjusting cognitive-motor interference. This dynamic modulation reduces temporal overlap with capacity-limited concurrent cognitive operations and thus could ensure that cognitive dual-tasking demands do not overly impair balance control.

Overall, these findings help to explain the interaction between cognitive processes and balance control. Our data suggests that balance control operates intermittently and that adjustments can be strategically reduced during periods of cognitive load. This interference is mostly driven by the temporal structure of the cognitive tasks and less by the specific cognitive processes that we examined in our study (i.e., memory consolidation and response selection). Once cognitive demands decrease, balance adjustments become more probable again.

Our findings can be interpreted in light of two possible mechanisms. One possibility is a passive bottleneck account, according to which cognitive and balance control share overlapping, capacity-limited processing streams (Johannsen et al., 2023). In this view, balance adjustments are suppressed or delayed simply because concurrent cognitive operations consume limited resources, creating a structural constraint (Pashler, 1994). However, our data are also consistent with a proactive regulation account, whereby balance control is strategically timed in anticipation of cognitive demands, such that adjustments are delayed or advanced to reduce cognitive-motor interference (Gawthrop et al., 2011; Lacour et al., 2008). This interpretation assumes that supramodal anticipatory mechanisms flexibly coordinate separate but interconnected streams of cognitive and balance control (Ivanenko & Gurfinkel, 2018).

We interpret our data pattern as evidence for a proactive regulation mechanism rather than a passively constrained regulation of balance control because the systematic reduction of adjustments around cognitively demanding phases suggests an adaptive postponement rather than a mere limitation of capacity. Nonetheless, the present data cannot conclusively distinguish between a passive bottleneck and a proactive regulation mechanism, and future research should employ paradigms with less predictable task timing and higher cognitive load to test whether balance adjustments are indeed strategically modulated in anticipation of cognitive demands.

### Limitations and future directions

Our experiments provide valuable insights into the interaction between cognitive dual-task demands and balance control, but several limitations should be noted. One key limitation was the inherent variability in trial length, particularly between short and long SOA trials but also between report and ignore trials, which might have influenced the observed effects on balance control. Ignore trials are much shorter and might not allow enough time for subjects to return to baseline in their balance control. Specifically, the difference in report and ignore trial length could be solved by bridging the time at the end of ignore trials to make them temporally more comparable to report trials, even though this might lead to other unwanted effects (i.e., if a very long waiting time occurs).

The absence of process-specific effects of SOA and task load might also be due to the relatively easy visual-vocal short-term memory task, with overall accuracy of around 98%. Introducing a more difficult memory task might occupy the memory-consolidation bottleneck for an even longer time, which could have potential to carry over to the balance domain. Yet this apparently easy task produced dual-task costs of around 100 ms.

The findings of this study are primarily generalisable to healthy young adults, as this population comprised our sample. While the results provide insights into cognitive-motor interference during dual-task performance, generalisation to older adults, children, or clinical populations with balance or cognitive impairments requires further investigation. Future research should examine whether similar patterns of balance adjustments occur in these groups to assess broader applicability.

Future studies could explore how other types of cognitive tasks, such as cognitive conflict or memory tasks, impact balance control in the range of sub-second time periods. In addition, it would be valuable to investigate balance control under less predictable temporal conditions, for example by varying the onset and timing between concurrent tasks, to examine whether and how anticipatory balance adjustments occur before cognitive processing begins. Moreover, varying the complexity and sequencing of tasks and exploring the mutual influence of balance and cognitive processes can be useful. Using non-graspable objects for the visual task would give more insights into possible effects independent of possibly activating motor codes (e.g., affordances, Tucker & Ellis, 1998). Completely avoiding visual stimuli could give insights into the effect of cognitive processes independent of visual information for balance adjustments.

In the current study, we used a cognitive dual-task paradigm which created a memory-consolidation bottleneck. Our results should act as a starting point to investigate other cognitive processes and their influence on balance control to extend our understanding of cognitive-motor interference. Implementing the novel analysis method could elucidate whether and at which stages of processing specific cognitive demands influence balance control.

## Conclusion

Overall, the event-related analysis of force-plate data provides insight into the complex interactions between cognitive processes and balance control during dual-task performance in the range of sub-second time periods which could not have been found with traditional approaches of aggregating force-plate data. The results underline the change of balance adjustments in response to cognitive demands and highlight the interference between cognitive and motor control in multitasking scenarios. We did not find that process-specific effects at the cognitive level carry over to the balance domain but a more general adaptation of balance adjustments before and during cognitive tasks. We assume that flexibility allows the balance control system to delay or advance balance adjustments, thereby reducing or adaptively adjusting cognitive-motor interference. This dynamic modulation can ensure that cognitive dual-tasking demands do not overly impair balance control. As our understanding of these interactions deepens, it may inform the development of interventions aimed at improving cognitive and physical performance in contexts where dual-tasking in combination with balance control is required.

## Data Availability

All data, analysis code, and research materials are available under these stable links: 10.23668/psycharchives.21433, 10.23668/psycharchives.21434, 10.23668/psycharchives.21435.

## Footnotes

1 Note that the term *control* in the balance control literature usually refers to the regulation of body stability, balance, and posture (Ivanenko & Gurfinkel, 2018). This term is related to a controller which is a concept often referring to a mechanism or system that makes the necessary adjustments to keep or achieve a specific state. In cognitive psychology, the term *control* is used in a broader sense and refers to mechanisms, functions, or processes which regulate thoughts, actions (possibly also motor control), and behaviors (Egner, 2017).

2 In addition, Koch and Rumiati (2006) manipulated compatibility of response sets across tasks. The visual object could require a response at the same compatible side (e.g., left) as the tone (e.g., also left) or the responses could mismatch (incompatible trial). They found shorter RTs in compatible compared to incompatible trials (which was replicated; Koch, 2009). However, the present study does not focus on this specific compatibility effect but on the generic dual-task interference effect.

3 In this text, the term *moment* always refers to its physical meaning (i.e., torque), and should not be confused with other uses of the word.

4 Including the site of testing as a between-subjects factor in the analysis revealed no significant effects.

5 Force-plate data were baseline-corrected using the first 200 ms of each trial. This procedure did not affect our dependent measure, as we computed variability, which is independent of absolute baseline shifts.

6 10.23668/psycharchives.21433, 10.23668/psycharchives.21434, 10.23668/psycharchives.21435.

7 For completeness, we assessed the role of compatibility of response sets across tasks (required response to the visual-vocal short-term memory task and required response to the auditory-manual RT task) in this dual-task paradigm. We found a compatibility effect (difference between compatible and incompatible trials) only at short SOA(1.8%; 33 ms) but not at long SOA (0.1%; -4 ms), *p*s $< .03$ for the interaction effects (SOA $\times$ compatibility) for both RTs and error rates. Yet, compatibility did not affect moment variability, so that we disregard this variable in the following.

8 For completeness, we assessed the role of compatibility of response sets across tasks in this dual-task paradigm. We only found a main effect of compatibility on error rates of the auditory-manual RT task, $F(1, 47) = 7.63$, $p = .008$, indicating higher error rates for incompatible trials (2.3%) than for compatible trials (1.7%). But there was no main significant effect of compatibility on RTs in the auditory-manual RT task, $F(1, 47) = 1.67$, $p = .203$. Interaction effects including compatibility were not significant with *F* $\le$ 2.55 and *p* $\ge$ .117.
